# Evaluation of the success of predicted dental changes with clear-aligner treatment: A pilot study

**DOI:** 10.1016/j.sdentj.2024.02.012

**Published:** 2024-02-24

**Authors:** Waleska Caldas, Fabiana Aparecida Bonin, João Lucas Moraes Piscinini, Camila Pereira Vianna, Roberto Hideo Shimizu, Larissa Carvalho Trojan

**Affiliations:** a Neodent, Curitiba, Brazil; b Curitiba, Brazil; c Ilapeo College, Curitiba, Brazil; d Straumann Group LLC, Irvine, USA

**Keywords:** Malocclusion, Orthodontic appliances, Clear aligners, Tooth movement techniques

## Abstract

**Objective:**

This pilot study concerned evaluation of the success of predicted dental changes in patients presenting with Class I malocclusions who were submitted to treatment aligners, using the superimposition.

**Methods:**

The digital models were superimposed and analyzed using 3DSlicer 5.0 software. Treatment and predicted changes regarding horizontal and vertical linear displacements, mesiodistal rotations, and incisor buccolingual tipping were quantified. The success rates were calculated by dividing the mean treatment change amount by the predicted change amount**.**

**Results:**

Lower-incisor intrusion was the most accurate of the predicted vertical displacements (86.96 %), and buccal expansion of upper canines (99.32 %) and mesial translation of the lower incisors (98.57 %) were the most accurate horizontal linear displacements. The predicted rotation was achieved with the highest accuracy for lower incisors (75.69 %). Incisor buccolingual tipping success rates ranged between 45.78 % and 69.31 %. Low accuracy of predicted changes was found for upper-molar extrusion (10.23 %) and constriction (8.91 %). However, minimal corrections in these directions were planned.

**Conclusions:**

Dental changes for all regions of maxillary and mandibular arches could be evaluated. High success rates were observed for most of the movements planned for ClearCorrect aligner therapy.

## 1 Introduction

Clear aligners are an alternative to conventional fixed appliances due to their aesthetic benefits, oral hygiene, and reduced visits to the orthodontist (Buschang et al., 2014, Bahammam and El-Bialy, 2023).

Three-dimensional superimposition of digital models is a way to measure tooth displacement (Garib et al., 2019). For the maxillary models, stable anatomical structures, such as the palatal rugae, are used as landmarks (Almeida et al., 1995b). For mandibular models, in the absence of a reference anatomical structure, untreated teeth are usually used (Almeida et al., 1995a). The disadvantage of this method is that it can only be applied when treatment planning includes not moving a certain group of lower teeth.

This study concerned evaluation of the success of predicted dental changes in patients presenting with Class I malocclusions who were submitted to treatment aligners, using the superimposition.

## 2 Materials and methods

This study was approved (#08697018.5.0000.8387). The inclusion criteria were five adult patients, healthy, compliant, and motivated, with Class I malocclusion. Exclusion criteria were gross gingival/periodontal problems, severe crowding, recent extraction, and tooth trauma. The patients’ median age was 30 years (min 23 - max 37). These patients need orthodontic treatment to resolve complaints of anterior crowding and tooth gaps. Four of them had underigone orthodontic treatment with fixed appliances (>2 years). A single experienced orthodontist at a private practice (Curitiba, Brazil) provided treatment from 2018 to 2019. Written consent was obtained from the sample. A summary of the patients’ characteristics and treatment protocol can be found in the supplementary material.

All subjects were submitted for orthodontic treatment with ClearCorrect aligners. Impressions were obtained using the 3Shape TRIOS intraoral scanner (3Shape A/S, Denmark) and addressed to the ClearComm platform for treatment planning. A lateral radiograph of the head and a panoramic radiograph were considered for the treatment.

In all cases, the treatment plan required the use of retention attachments on the buccal surfaces of various teeth depending on the tooth movement required. Attachments presented rectangular shape and dimensions of 1 × 2 × 3 mm (thickness, width, length). Vertical attachments were used to favor tilting and translation.

Inter-proximal reduction (IPR) was necessary in some cases to resolve tooth crowding. Case 5 had only the upper teeth moved. Aligners were replaced with a mean interval of 2.4 ± 0.21 weeks. A slightly longer period was sometimes needed to allow for the occurrence of more complex movements. Patients were instructed to wear the aligners for 22 h per day. The median number of steps was 13 aligners, with a minimum of 10 and a maximum of 18 aligners.

The digital models were stored as “stl” files − 3D Slicer 5.0 software (https://www.slicer.org).

The steps for the superimposition of digital models were performed following the methods Garib et al.(Garib et al., 2019) established (palatal rugae).

**Fig. 1:**
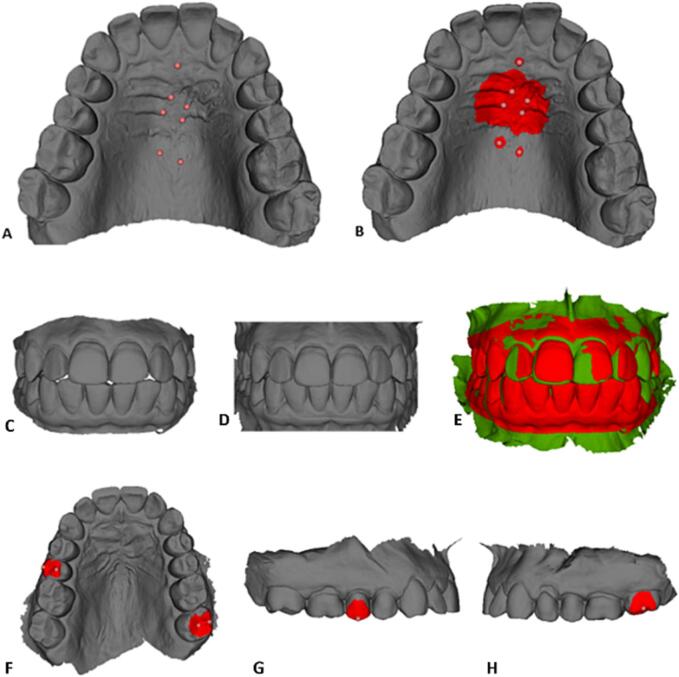
(A) Seven landmarks’ registration on the palatal rugae (Garib et al., 2019). (B) Regions of interest used for ROI registration. (C, D, and E) are from Case 1. (C) Pretreatment and (D) posttreatment. (E) Superimposition (pretreatment in red and posttreatment in green). (F, G, and H) are examples of regions of interest.

For orientation and approximation, corresponding landmarks were placed at the posterior limit of the incisive papilla, at the medial edges of the second palatal rugae, and at the medial edges of the third rugae. Two additional landmarks were projected 10 mm distal to the medial edge of the third-rugae landmarks (Fig. 1A). These landmarks were used to define regions of interest (ROIs). Each ROI’s dimension was defined as the radius of 5 vertices of the model meshes for the incisive papilla and the third-rugae projection landmarks. The medial edges of the second and third rugae were assigned with ROIs with a radius of 30. The regions of interest around the landmarks were in this way never extended close to the tooth region (Fig. 1B). The Slicer “CMF registration” tool then superimposed the posttreatment maxillary model relative to the pretreatment model by matching the coordinates of the corresponding ROIs (ROI registration).

### 2.1 Superimposition pre- and posttreatment maxillary and mandibular models

**Fig. 2:**
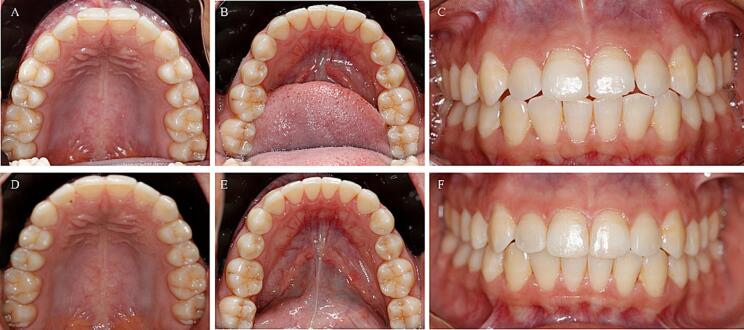
Clinical aspects pretreatment (A, B, and C) and posttreatment (D, E, and F) of Case 1.

The superimposition of the pre and post-treatment mandible was made in occlusal relation (Fig. 1C and 1D) to the maxilla, at which point it is as if the two models (maxilla and mandible) become one 3D model. The matrices generated from the maxillary models’ superimposition process were applied to the corresponding pretreatment and posttreatment mandibular models at all steps of the process, changing the position and orientation of the 3D models and allowing for their superimposition (Fig. 1F, 1G, and 1H). Patients’ initial and final predicted models were retrieved from ClearComn already superimposed (see Fig. 2).

### 2.2 Quantification of changes

Once the pretreatment, posttreatment, and predicted models were in their final positions, landmarks were equally placed in all of them to allow for quantification of treatment and predicted changes.

The landmarks were placed in maxillary and mandibular models as follows:

• At the tip of the mesiobuccal cusp of the maxillary first and second molars, the mesiobuccal groove of the mandibular first molar, and the central groove of the mandibular second molar; at the tip of the buccal cusp of all premolars and canines; and on the mesial angle of the incisal edge of all incisors to allow for the measurement of horizontal and vertical linear displacements.
• At the most mesial and distal points of all teeth, an occlusal perspective to allow for quantification of mesiodistal rotations.
• At the center of the incisal edge and the deepest point of the cervical level of all incisors to allow for evaluation of buccolingual tipping.

The x, y, and z coordinates of the landmarks related to linear displacements were obtained for the pretreatment and posttreatment models as well as initial and final predicted models using the Fiducial Registration Wizard tool in 3D Slicer. The differences between coordinates were then calculated to determine the number of achieved and predicted changes.

The Q3DC tool was applied to measure the angle formed between the lines that connected the mesial and distal landmarks (mesiodistal rotations) as well as the incisal and cervical landmarks (buccolingual tipping) of each tooth between each pair of models (pre- and posttreatment/initial and final predicted models).

One calibrated investigator made all measurements and repeated them 30 days thereafter. The random errors were calculated using Dahlberg’s formula (Se^2^ = ∑d^2^/2n), and the systematic errors were evaluated with dependent t-tests, with the level of significance set at P < 0.05.

All descriptions were described by means and standard deviations and performed separately for each type and direction of tooth displacement. The success rates were calculated by dividing the mean amount of treatment changes by the mean number of predicted changes.

## 3 Results

Random errors were within the acceptable limits (not greater than 0.2 mm or 0.5 degrees), and there was no significant systematic error. All patients were successfully treated with a normal occlusion, with ideal overjets and overbites. The mean treatment time was 9.75 months. The following measures can be found in the supplementary material.

Lower-incisor intrusion was the most accurate of the predicted vertical displacements, with a mean success rate of 86.96 %, followed by upper-molar intrusion, with a mean success rate of 84.86 %. Meanwhile, upper-molar extrusion was the least accurate (10.23 % success rate).

When buccolingual linear displacements were evaluated, buccal expansion of the upper and lower canines were the most accurate movements, presenting mean success rates of 99.32 % and 97.87 %, respectively. On the other hand, upper-molar constriction was the least successful (8.91 %).

As for mesiodistal linear displacements, it was demonstrated that mesial translation of the lower incisors and distal translation of the lower canines presented the highest success rates, with a mean of 98.57 % and 82.78 %, respectively. Distalization of the lower premolars, on the other hand, presented the lowest success rate (43.68 %). As for the angular corrections, the predicted rotation was achieved with the highest accuracy for the lower incisors and lower premolars (75.69 % and 67.04 %, respectively) and with less predictability for the lower canines (37.55 %).

As for the angular corrections, the predicted rotation was achieved with the highest accuracy for the lower incisors and lower premolars (75.69 % and 67.04 %, respectively) and with less predictability for the lower canines (37.55 %).

The success rates of incisor buccolingual tipping ranged between 45.78 % (buccal tipping of upper incisors) and 69.31 % (buccal tipping of the lower incisors).

Below is an example of the final aspect.

## 4 Discussion

The mandibular torus is a reference for three-dimensional superimposition. However, it can only be applied to a limited number of patients (An et al., 2015, Choi et al., 2018). Therefore, due to the absence of another reliable anatomical structure, some studies have reported the use of untreated teeth as a reference for mandibular superimposition. This method, however, cannot be applied in cases in which all teeth have been moved (Chen et al., 2011, Vasilakos et al., 2017), which is why this study included a different method.

Regarding palatal rugae, it seems clear that if pre- and posttreatment models are obtained with the maxilla and mandible in an occlusal relationship, the maintenance of this interrelation during the maxillary superimposition process can equally allow for reliable superimposition of mandibular models. The presented method allowed for the evaluation of treatment changes for the anterior and posterior lower teeth.

Regarding success, vertical movements have been reported to be the most difficult to achieve (Robertson et al., 2020). In the present study, the mean success rate was 67.7 % for tooth intrusion and 50.48 % for extrusion. Although those are relatively low values, they are still higher than the mean rates reported in previous studies, which range from 41.3 % to 46.6 % for intrusion and 18.3 % to 24.5 % for extrusion (Robertson et al., 2020). The extrusion of the upper molars was the least accurate vertical movement, with a 10.23 % success rate. Canines are more difficult to move accurately with aligners without the aid of intermaxillary elastics or other auxiliary devices, often leading to low accuracy (Buschang et al., 2014). Accordingly, in the present study, extrusion of upper canines was the second-least accurate vertical movement (35.72 %).

Buccal expansion of upper and lower canines was the most accurate of the buccolingual linear displacements (mean success rates of 99.32 % and 97.87 %, respectively). It has been reported that clear-aligner therapy is effective and accurate for anterior-tooth horizontal displacements. On the other hand, constriction of molars can be difficult to achieve, even with conventional fixed orthodontic therapy (Lombardo et al., 2017). These were not deemed necessary for any of the present study cases because only minor corrections in this direction were needed. Still, upper and lower molar constriction were the least accurate (8.91 % and 14.29 %, respectively). Similar outcomes have been reported, and statistically significant differences were found between predicted and achieved movements for upper premolar and molar buccolingual linear displacements(Robertson et al., 2020, Grünheid et al., 2017).

Mesial translation of the lower incisors and distal translation of the lower canines presented the highest success rates for mesiodistal changes (98.57 % and 82.78 %, respectively), and distalization of the lower premolars presented the lowest rate (43.68 %).

The predicted rotation was achieved with the highest accuracy for the lower incisors and lower premolars (75.69 % and 67.04 %, respectively) and with less predictability for lower canines (37.55 %). The rotational movements present low predictability and are influenced by tooth morphology, which significantly affects the forces and moments applied by aligners (Buschang et al., 2014, Barreda et al., 2017). Therefore, the low accuracy presented in the present study for the rotation of mandibular canines, which are round teeth and present large root surface areas, was expected, as observed in another study (Cortona et al., 2020).

As for buccolingual crown tipping, only upper and lower incisors were evaluated because for this type of angular measurement, only this region has been shown to generate reliable results for measurement using only digital model analysis (Kravitz et al., 2008).

Finally, IPR procedures should be considered during treatment planning because some studies have reported that the combination of attachments and IPR helps achieve rotational corrections, especially of canines (Kravitz et al., 2008, Alam et al., 2023).

Because this was a pilot study with only five patients, replication of this method can be considered a limitation. In the future, more studies should be conducted with larger samples and involving more complex malocclusion corrections to confirm the present findings.

## 5 Conclusions

Dental changes for all regions of arches could be evaluated. Treatment with ClearCorrect aligners showed a high success rate for the proposed treatment, demonstrating high performance for its purpose, as shown for each type of tooth displacement.

## Funding

This work was financially supported by ClearCorrect (Straumann Group).

## CRediT authorship contribution statement

**Waleska Caldas:** Methodology, Formal analysis, Manuscript original draft. **Fabiana Aparecida Bonin:** Patient treatment, Investigation. **João Lucas Moraes Piscinini:** Data curation. **Camila Pereira Vianna:** Manuscript original draft. **Roberto Hideo Shimizu:** Supervision, Manuscript review & editing. **Larissa Carvalho Trojan:** Conceptualization, Project administration, Supervision, Manuscript review & editing.

## Declaration of competing interest

The authors declare the following financial interests/personal relationships which may be considered as potential competing interests: Fabiana Aparecida Bonin reports a relationship with ClearCorrect (Straumann Group) that includes: employment. If there are other authors, they declare that they have no known competing financial interests or personal relationships that could have appeared to influence the work reported in this paper.

João Lucas Moraes Piscinini reports a relationship with ClearCorrect (Straumann Group) that includes: employment. If there are other authors, they declare that they have no known competing financial interests or personal relationships that could have appeared to influence the work reported in this paper.

Larissa Carvalho Trojan reports a relationship with ClearCorrect (Straumann Group) that includes: employment. If there are other authors, they declare that they have no known competing financial interests or personal relationships that could have appeared to influence the work reported in this paper.

Roberto Hideo Shimizu reports a relationship with ClearCorrect (Straumann Group) that includes: employment. If there are other authors, they declare that they have no known competing financial interests or personal relationships that could have appeared to influence the work reported in this paper.

Waleska Caldas reports a relationship with ClearCorrect (Straumann Group) that includes: employment. If there are other authors, they declare that they have no known competing financial interests or personal relationships that could have appeared to influence the work reported in this paper.

Camila Pereira Vianna reports a relationship with ClearCorrect (Straumann Group) that includes: employment. If there are other authors, they declare that they have no known competing financial interests or personal relationships that could have appeared to influence the work reported in this paper.
