# Supplementary material for: Evaluation of the success of predicted dental changes with clear-aligner treatment: A pilot study
Source: Saudi Dent J. 2024 Feb 24;36(5):708–11. doi: 10.1016/j.sdentj.2024.02.012 (PMC11096612; doi:10.1016/j.sdentj.2024.02.012)
Supplement: Supplementary Data 1 [file mmc1.docx]

**SUPPLEMENTARY MATERIAL**

| Table 1 - Patient Characteristics and Treatment Protocol | | | | | | | | | | | |
| --- | --- | --- | --- | --- | --- | --- | --- | --- | --- | --- | --- |
| Subjects/ cases | Gender | Age (years) | Overbite (mm) | Overjet (mm) | Space Analysis  (mm) | | Steps | Attachments | | IPR | |
|  |  |  |  |  | maxilla | mandible |  | maxilla | mandible | maxilla | mandible |
| 1 | Male | 37 | 0.7 | 0.8 | -3.54 | -2.6 | 18 | 11,21 | 34 | 0.6mm | - |
| 2 | Female | 28 | 2.1 | 2.8 | -1.17 | 0 | 10 | 13,12,11,21 | - | 1.2mm | - |
| 3 | Female | 36 | 3.2 | 2.2 | -1 | -3 | 15 | 23 | 43 | 0.6mm | 2.7mm |
| 4 | Male | 30 | 1.8 | 1.2 | 0 | -3 | 13 | 15,12,22,25 | 45,42,32,35 | 0.3mm | 1.8mm |
| 5 | Female | 23 | 2.9 | 2.0 | -1.7 | - | 11 | 12,22 | - | 0.6mm | - |

| Table 2 - Mean changes and success of vertical displacements and buccolingual displacements (mm). | | | | | | | | | | | | | | | | |
| --- | --- | --- | --- | --- | --- | --- | --- | --- | --- | --- | --- | --- | --- | --- | --- | --- |
|  | Vertical Displacements | | | | | | | | Buccolingual Displacements | | | | | | | |
|  | Intrusion | | | | Extrusion | | | | Lingual constriction | | | | Buccal expansion | | | |
|  | n | Mean  (mm) | SD* | Mean  Success  (%) | n | Mean  (mm) | SD* | Mean  Success  (%) | n | Mean  (mm) | SD* | Mean  Success  (%) | n | Mean  (mm) | SD* | Mean  Success  (%) |
| Upper Incisors | 14 | 0.17 | 0.17 | 45.21 | 6 | 0.39 | 0.33 | 65.72 | 13 | 0.45 | 0.48 | 59.65 | 7 | 0.24 | 0.19 | 41.64 |
| Upper Canines | 5 | 0.22 | 0.15 | 37.83 | 5 | 0.17 | 0.27 | 35.72 | 8 | 0.21 | 0.25 | 44.03 | 2 | 0.08 | 0.09 | 99.32 |
| Upper Premolars | 13 | 0.48 | 0.34 | 73.84 | 7 | 0.16 | 0.11 | 49.46 | 12 | 0.18 | 0.25 | 24.30 | 8 | 0.45 | 0.17 | 94.31 |
| Upper Molar | 12 | 0.58 | 0.49 | 84.86 | 8 | 0.05 | 0.10 | 10.23 | 10 | 0.06 | 0.15 | 8.91 | 10 | 0.52 | 0.32 | 97.78 |
| Lower Incisors | 8 | 0.58 | 0.31 | 86.96 | 8 | 0.24 | 0.14 | 67.03 | 11 | 0.76 | 0.54 | 70.45 | 5 | 0.73 | 0.69 | 86.15 |
| Lower Canines | 5 | 0.42 | 0.08 | 84.40 | 3 | 0.13 | 0.18 | 38.07 | 5 | 0.39 | 0.27 | 75.19 | 3 | 0.55 | 0.18 | 97.87 |
| Lower Premolars | 12 | 0.24 | 0.21 | 59.78 | 4 | 0.24 | 0.16 | 69.97 | 5 | 0.42 | 0.31 | 86.86 | 11 | 0.48 | 0.22 | 87.06 |
| Lower Molar | 13 | 0.23 | 0.19 | 66.11 | 3 | 0.30 | 0.14 | 67.66 | 7 | 0.02 | 0.06 | 14.29 | 9 | 0.51 | 0.31 | 89.58 |
| Total | 82 | 0.36 | 0.24 | 67.37 | 44 | 0.21 | 0.18 | 50.48 | 71 | 0.31 | 0.29 | 47.96 | 55 | 0.45 | 0.27 | 86.71 |
| *SD = Standard Deviation | | | | | | | | | | | | | | | | |

| Table 3 - Mean changes and success of mesio-distal displacements (mm). | | | | | | | | |  |  |  |  |
| --- | --- | --- | --- | --- | --- | --- | --- | --- | --- | --- | --- | --- |
|  | Distalization | | | | Mesialization | | | | Rotation | | | |
|  | n | Mean (mm) | SD* | Mean success (%) | n | Mean (mm) | SD* | Mean success (%) | n | Mean (°) | SD* | Mean success (%) |
| Upper Incisors | 9 | 0.39 | 0.26 | 67.87 | 11 | 0.32 | 0.38 | 47.66 | 20 | 2.92 | 2.71 | 54.21 |
| Upper Canines | 7 | 0.42 | 0.37 | 57.41 | 3 | 0.36 | 0.19 | 50.87 | 10 | 3.87 | 2.17 | 72.02 |
| Upper Premolars | 11 | 0.31 | 0.35 | 47.57 | 9 | 0.39 | 0.20 | 74.51 | 20 | 2.31 | 2.20 | 52.26 |
| Upper Molars | 8 | 0.49 | 0.42 | 57.65 | 12 | 0.42 | 0.25 | 73.82 | 20 | 1.69 | 1.56 | 56.51 |
| Lower Incisors | 12 | 0.37 | 0.35 | 64.39 | 4 | 0.84 | 0.04 | 98.57 | 16 | 4.14 | 3.19 | 75.69 |
| Lower Canines | 6 | 0.86 | 0.50 | 82.78 | 2 | 0.23 | 0.07 | 62.16 | 8 | 3.55 | 4.24 | 37.55 |
| Lower Premolars | 7 | 0.31 | 0.37 | 43.68 | 9 | 0.23 | 0.25 | 55.14 | 16 | 3.23 | 2.84 | 67.04 |
| Lower Molars | 5 | 0.32 | 0.23 | 52.71 | 11 | 0.27 | 0.23 | 63.83 | 16 | 1.99 | 2.14 | 56.56 |
| Total | 65 | 0.43 | 0.35 | 59.26 | 61 | 0.38 | 0.20 | 65.82 | 126 | 2.96 | 2.63 | 58.98 |
| *SD = Standard Deviation | | | | | | | | |  |  |  |  |

| Table 4 – Mean changes and success of buccolingual tipping (°). | | | | | | | | |
| --- | --- | --- | --- | --- | --- | --- | --- | --- |
|  | Buccolingual Tipping | | | | | | | |
|  | Lingual Tipping | | | | Buccal Tipping | | | |
|  | n | Mean (°) | SD* | Mean success (%) | n | Mean | SD* | Mean success (%) |
| Upper Incisors | 11 | 3.07 | 2.85 | 60.26 | 9 | 0.62 | 1.05 | 45.78 |
| Lower Incisors | 11 | 1.39 | 1.36 | 57.35 | 5 | 3.40 | 3.23 | 69.31 |
| Total | 22 | 2.23 | 2.11 | 58.80 | 14 | 2.01 | 2.14 | 57.55 |
| *SD = Standard deviation | | | | | | | | |
